# Tamoxifen plus tegafur-uracil (TUFT) versus tamoxifen plus Adriamycin (doxorubicin) and cyclophosphamide (ACT) as adjuvant therapy to treat node-positive premenopausal breast cancer (PreMBC): results of Japan Clinical Oncology Group Study 9404

**DOI:** 10.1007/s00280-014-2545-2

**Published:** 2014-07-24

**Authors:** Tadahiko Shien, Hiroji Iwata, Takashi Fukutomi, Kenichi Inoue, Kenjiro Aogi, Takayuki Kinoshita, Jiro Ando, Seiki Takashima, Kenichi Nakamura, Taro Shibata, Haruhiko Fukuda

**Affiliations:** 1 Department of Breast and Endocrine Surgery, Okayama University Hospital, Okayama, 700-8558 Japan; 2 Department of Breast Oncology, Aichi Cancer Center Hospital, Nagoya, 464-8681 Japan; 3 Department of Breast Surgery, National Cancer Center Hospital, Tokyo, 104-0045 Japan; 4 Department of Medical Oncology, Saitama Cancer Center, Saitama, 362-0806 Japan; 5 Department of Surgery, National Hospital Organization Shikoku Cancer Center, Matsuyama, 791-0280 Japan; 6 Department of Breast Surgery, Tochigi Cancer Center, Utsunomiya, 320-0834 Japan; 7 JCOG Data Center, Multi-institutional Clinical Trial Support Center, National Cancer Center, Tokyo, 104-0045 Japan

**Keywords:** Breast cancer, Adjuvant treatment, Node-positive, Premenopausal

## Abstract

**Purpose:**

A prospective randomized clinical trial was conducted to evaluate the efficacy of tamoxifen plus doxorubicin and cyclophosphamide compared to tamoxifen plus tegafur-uracil as an adjuvant therapy to treat node-positive premenopausal breast cancer (PreMBC).

**Methods:**

Eligibility criteria included pathologically node-positive (*n* = 1–9) preMBC with curative resection, in stages I–IIIA. Patients were randomized to receive either tamoxifen 20 mg/day plus tegafur-uracil 400 mg/day (TU) for 2 years or six courses of a 28-day cycle of doxorubicin 40 mg/m^2^ plus cyclophosphamide 500 mg/m^2^ on day 1 along with tamoxifen (ACT) given for 2 years as adjuvant therapy. Primary endpoint was overall survival (OS), and secondary endpoint was recurrence-free survival (RFS).

**Results:**

In total, 169 patients were recruited (TU arm 87, ACT arm 82) between October 1994 and September 1999. The HR for OS was 0.76 (95 % CI 0.35, 1.66, log-rank *p* = 0.49) and that for RFS was 0.77 (95 % CI 0.44, 1.36, log-rank *p* = 0.37), with ACT resulting in a better HR. The 5-year OS was 79.7 % for patients in the TU arm and 83 % for those in the ACT arm. The 5-year RFS was 66.1 % for patients in the TU arm and 70.6 % for those in the ACT arm. A higher proportion of patients in the ACT arm experienced grade 3 leucopenia (0 % in the TU arm, 4 % in the ACT arm).

**Conclusions:**

There were no significant differences in the efficacy of TU and ACT as adjuvant therapy.

## Introduction

Progression-free survival and overall survival have been improved according to the development of postoperative adjuvant therapy using drugs based on clinical trials. Prior to the 1980s, cyclophosphamide, methotrexate, and fluorouracil (CMF) therapy was the standard therapy, but development of Adriamycin in the 1990s indicated that Adriamycin might surpass CMF in terms of prolonging prognosis. Prior to the 1990s, oral anticancer agents became the standard therapy since they were thought to cause fewer adverse events in Japan.

Combined administration of oral fluoropyrimidine plus tamoxifen for 2 years postoperatively was reported to result in a high 5-year survival of 91 % for patients with Stage II breast cancer and 78 % for those with Stage III breast cancer [1, 2], and combined administration of oral fluoropyrimidine plus tamoxifen was reported to diminish QOL less [1]. The criteria for determination of estrogen receptor (ER) status at the time differed from the current criteria, and tamoxifen was supposed to be less efficacious in ER-negative patients. However, tamoxifen was administered regardless of the patient’s ER status in general. Moreover, the form of administration was typically in combination with an anticancer agent including chemotherapy and hormone therapy. This study was planned within this context.

Current postoperative drug therapy to treat breast cancer is often chosen depending on the breast cancer subtype, which is determined based on panels for markers such as ER, HER2, and Ki67 [2]. This selection is based on predicted drug efficacy. The fact that lymph node metastasis is a prognostic factor was true when this trial began and it remains true today. When numerous lymph node metastases are noted, standard therapy is the administration of anthracycline and taxane, regardless of the cancer subtype. This study sought to assess the superiority of Adriamycin and cyclophosphamide (AC) + tamoxifen (ACT regimen) over oral tegafur-uracil (UFT) + tamoxifen (TU regimen), which was the standard therapy in Japan when the trial began, as a postoperative adjuvant therapy to treat premenopausal breast cancer in patients who were histopathologically confirmed to have lymph node metastasis. This trial also sought to determine whether all patients with node-positive breast cancer needed to be administered anthracycline or whether administration of oral fluoropyrimidine was sufficient.

## Patients and methods

### Eligibility and excluding criteria

Premenopausal female patients over the age of 15 with Stage I–IIIa breast cancer were eligible for this study. All patients had to have undergone curative mastectomy with axillary node dissection, and a histological examination had to reveal involvement of 1–9 axillary nodes. Other eligibility criteria were a World Health Organization (WHO) performance status of 0–1, adequate bone marrow and liver and kidney function, and no evidence of metastasis. Patients who received previous systemic treatment for breast cancer were excluded. The informed consent of each patient was obtained before study participation.

### Planned treatment schedules

All patients randomized to TU or ACT regimen. For patients in the TU arm, tamoxifen (20 mg/day) and UFT (400 mg/day) were administered for a maximum of 2 years in all patients. For patients in the ACT arm, Adriamycin (40 mg/m^2^ intravenously) and cyclophosphamide (500 mg/m^2^ intravenously) were administered on day 1 every 28 days. This cycle was repeated six times. Tamoxifen (20 mg/day) was administered for a maximum of 2 years in all patients, regardless of hormonal receptor status.

Randomization was done using the minimization method, and the arms were balanced with regard to ER and progesterone receptor (PR) status (either one positive (>10 %) versus both negative and unknown), HER2 status (positive versus negative or unknown), number of metastatic nodes (1–3 versus 4–9), and institution.

### Patient assessment

Initial workup included medical history, tumor assessment, physical examination, routine hematology and chemistry test, chest radiography, liver ultrasonography, and a bone scan. Hematology and chemistry tests, tumor marker measurements, and urinalysis were repeated monthly. To check for distant metastasis, a chest radiography and liver ultrasonography were performed every 6 months, a bone scan was performed every year, and bilateral mammography was performed every 2 years. Hematological disorders and toxicity were evaluated according to the Toxicity Grading Criteria of the Japan Clinical Oncology Group (JCOG) [3] and were recorded on case report forms.

### Study endpoint

The primary endpoint of this study was overall survival (OS), and the secondary endpoint was recurrence-free survival (RFS). OS was defined as the time from randomization to death from any cause, and it was censored as of the date of final follow-up. RFS was defined as the time from randomization to either the first incidence of recurrence or death from any cause, and it was censored as of the date of final follow-up. OS and RFS were evaluated according to hormone receptor status (either ER- or PR-positive versus both ER- and PR-negative or unknown) in subgroup analyses. In addition, the safety of treatment was evaluated.

### Statistical analysis plan

If patients treated with ACT had a significantly longer OS than patients treated with TU, then ACT would be recommended as the new standard treatment. The estimated 5-year OS of these patients is commonly 64–88 % [4–6]. A total of 342 patients were needed to detect a prolongation of the 5-year OS from 75 % for patients in the TU arm to 85 % for patients in ACT arm with an 80 % power and a two-sided alpha of 5 %. Considering some patients potentially lost to follow-up, the sample size was set at 400 patients in total. The planned study period was originally 2 years for recruitment and an additional 5 years for follow-up. Due to the slow recruitment, the protocol was revised to extend the recruitment period, and the sample size was revised to 330 patients with a recruitment period of 5 years. OS was analyzed for all randomized patients and RFS for randomized patients excluding a patient with bone metastasis at the registration. OS and RFS were estimated using the Kaplan–Meier method, and curves were compared using a log-rank test. Hazard ratios of treatment effects were estimated by a Cox regression model. All analyses were based on intent to treat. All statistical analyses were performed using SAS release 8.2 (SAS Institute, Cary, NC).

### Interim analysis and monitoring plan

An interim analysis was to be performed when half of the total number of patients was enrolled. The JCOG Data and Safety Monitoring Committee (DSMC) independently reviewed the interim analysis report, and premature termination of the trial could be considered at that stage. In-house interim monitoring was performed by the JCOG Data Center to ensure data submission, patient eligibility, protocol compliance, safety, and on-schedule study progress. The monitoring reports were submitted to and reviewed by the DSMC every 6 months.

## Results

This study began in 1994. At an interim analysis on June 1999, patient recruitment was so slow that the DSMC recommended terminating patient recruitment or continuing but changing the primary endpoint to RFS. Furthermore, a consensus meeting in St. Gallen in 1997 deemed that administering tamoxifen to hormone receptor-negative patients was ethically unacceptable [7]. Therefore, recruitment of patients was terminated pursuant to suggestions from the JCOG DSMC.

**Fig. 1:**
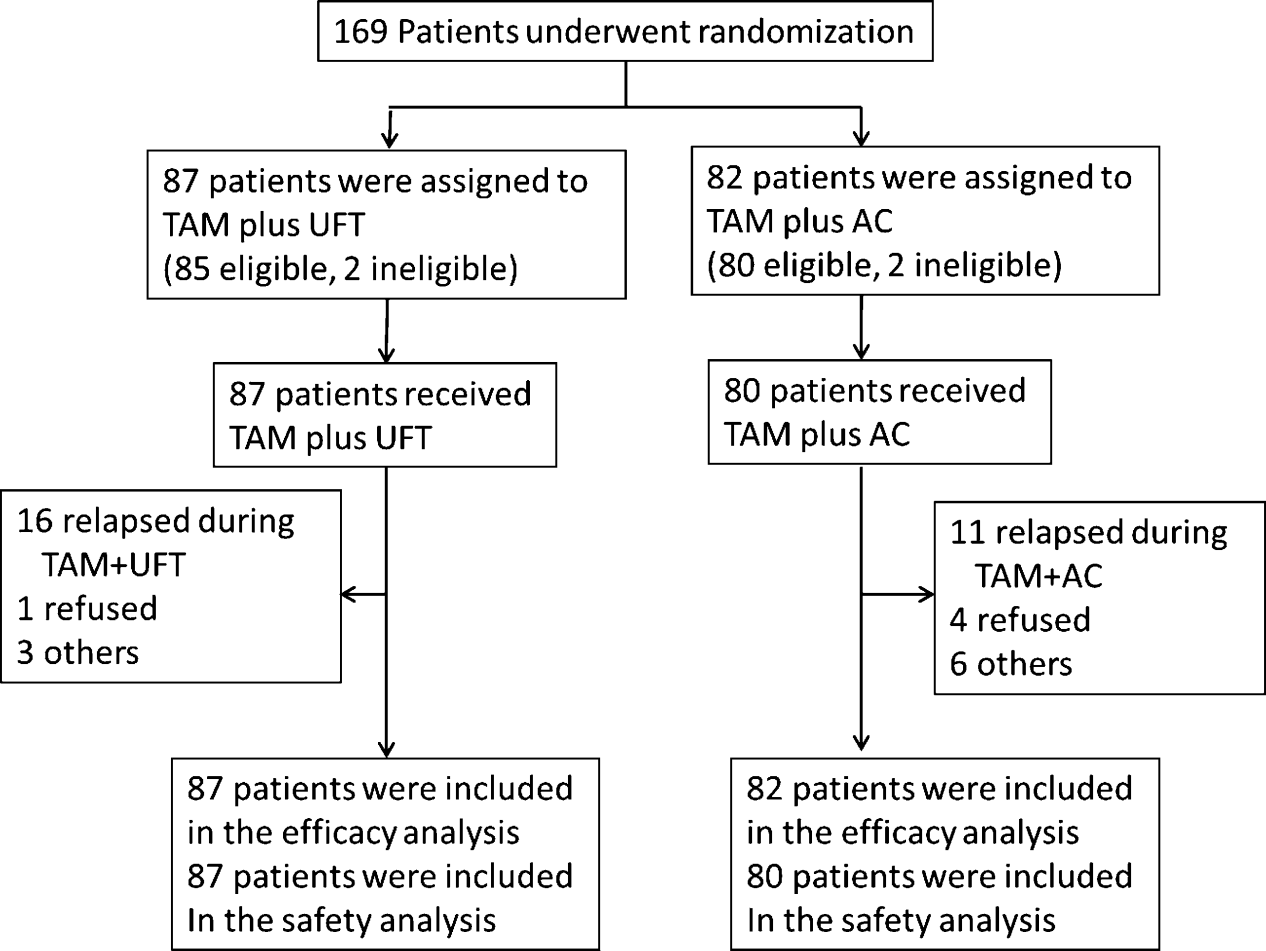
Trial profile of Japan Clinical Oncology Group study, JCOG 9404

In total, 169 patients were recruited and randomly assigned (Fig. 1). Four patients were ineligible because two were enrolled after starting protocol treatment, one had been diagnosed with bone metastasis, and the other was postmenopausal before recruitment, but these patients were included in the analysis. The two groups had highly similar baseline characteristics (Table 1). The median age was 46 years (30–56 years). One hundred and seventeen patients (69.2 %) had node metastases involving 1–3 nodes, while 52 (30.8 %) had node metastases involving 4–9 nodes. There were 59 patients (34.9 %) with ER- or PR-tumors, including patients with an unknown hormone status. Most patients (95.3 %) underwent total or radical mastectomy. Eighty-seven patients were assigned to the TU arm, and 82 patients were assigned to the ACT arm. Patient’s diagram was shown in Fig. 1. The protocol treatment in the TU arm was completed by 75 of 87 patients (86.2 %), and the protocol treatment in the ACT arm was completed by 66 of 82 patients (80.5 %).

**Table 1:** Patient characteristics

Characteristics	TU (*n* = 87)	ACT (*n* = 82)
Age (year)		
Median	47	45
Range	31–55	30–56
No. of positive axillary nodes		
1–3	59	58
4–9	28	24
ER and/or PgR		
Negative/unknown	29	30
Positive	58	52
HER2		
Negative/unknown	70	63
Positive	17	19
Stage		
I	12	12
II	58	60
IIIA	17	11
Operation		
Radical mastectomy	1	6
Total mastectomy	81	73
Partial resection	5	3

### Survival

**Fig. 2:**
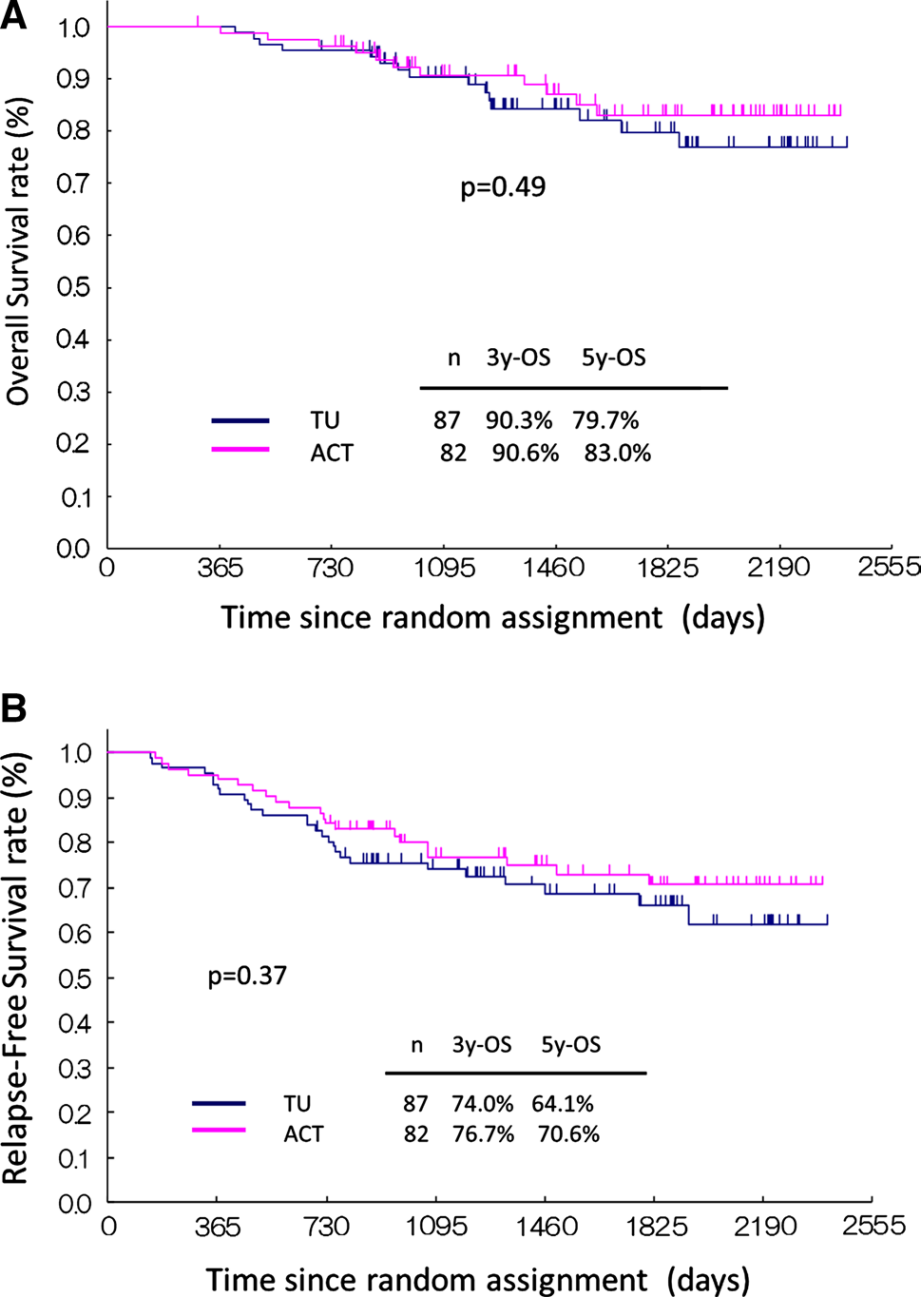
Kaplan–Meier curves of overall survival (**a**) and relapse-free survival (**b**) for node-positive breast cancer patients treated with tamoxifen plus tegafur-uracil or tamoxifen with anthracycline and cyclophosphamide

There were no significant differences in OS for patients in the two arms (*p* = 0.494, hazard ratio 0.76, 95 % confidence interval [CI] 0.35–1.66) (Fig. 2a). The 3- and 5-year OS were 90.3 and 79.7 % for patients in the TU arm and 90.6 and 83.0 % for patients in the ACT arm, respectively. There were no significant differences in RFS for patients in the two arms (*p* = 0.37, HR: 0.77, 95 % CI 0.44–1.36) (Fig. 2b). The 3- and 5-year RFS were 74.0 and 66.1 % for patients in the TU arm and 76.7 and 70.6 % for patients in the ACT arm, respectively.

**Fig. 3:**
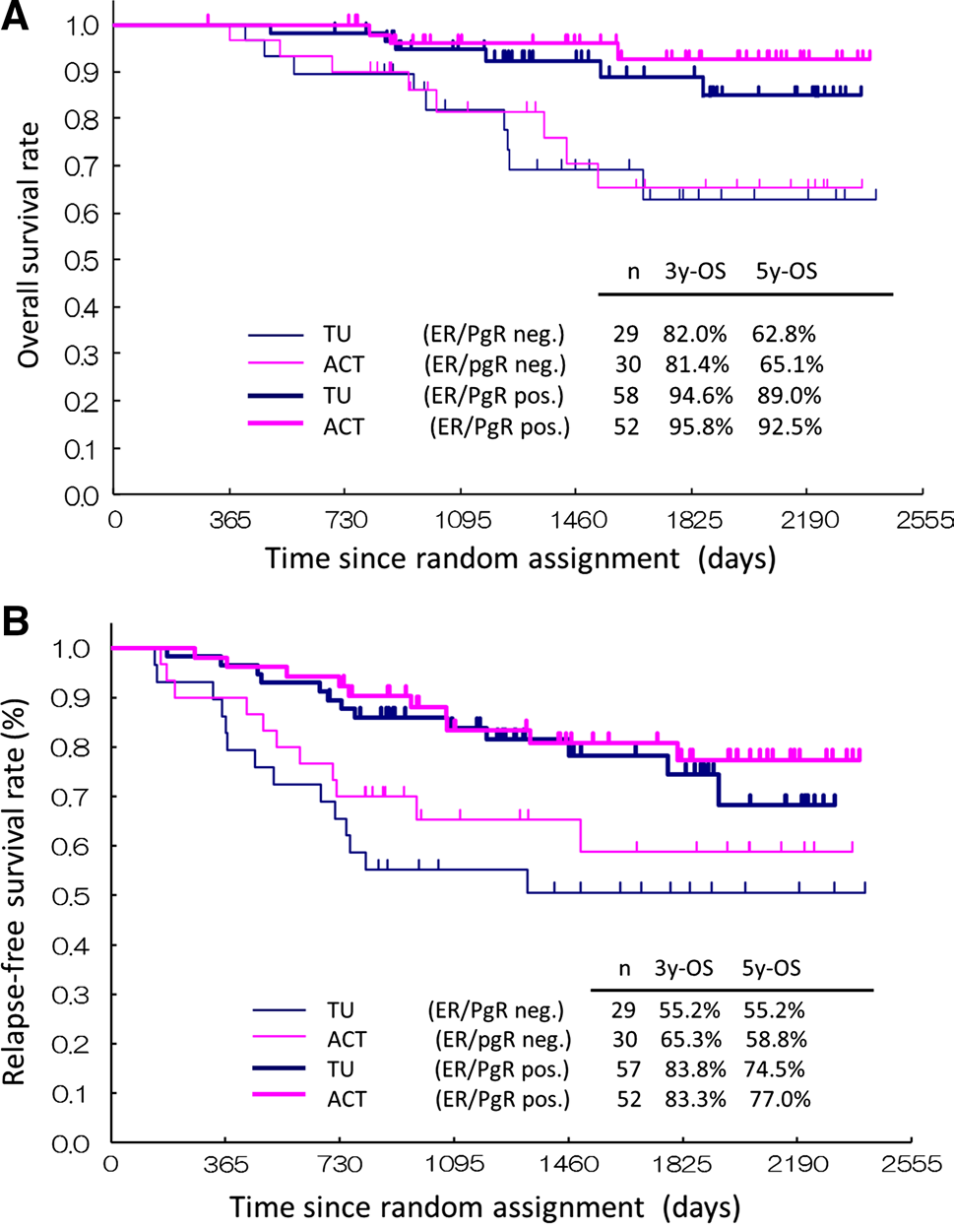
Kaplan–Meier curves of overall survival (**a**) and relapse-free survival (**b**) for node-positive breast cancer patients treated with tamoxifen plus tegafur-uracil or tamoxifen with anthracycline and cyclophosphamide according to estrogen receptor (ER) and progesterone receptor (PgR) status

Subgroup analysis was performed according to hormone receptor status. There were 57 patients (65.5 %) who were ER+ and/or PR+ in the TU arm and 52 (63.4 %) in the ACT arm. The OS curve is shown in Fig. 3a. Both ER- and PR-negative patients had a worse prognosis than ER-positive patients. However, patients in the TU and ACT arms had a similar OS, regardless of hormone status. Both ER- and PR-negative patients in the TU arm had a relatively shorter RFS than those in the ACT arm (Fig. 3b). There were no differences in the RFS of ER+ and/or PR+ patients in both arms.

### Safety profile

Safety profiles are listed in Table 2A and B. Only one patient was observed grade 4 adverse event (GPT elevation) in the TU arm. This event was diagnosed at 35th day after the start of TU, and once the administration of UFT was halted, GPT decreased to normal levels. A higher proportion of patients in the ACT arm had a lower white blood cell count that was rated grade 3 (0 % in the TU arm, 3.8 % in the ACT arm), and a higher proportion of patients in the TU arm had elevated total bilirubin, GOT, and GPT that were rated grade 3 (12.6, 2.3, and 2.3 % in the TU arm, 0, 1.3, and 1.3 % in the ACT arm) and lower hemoglobin (3.4 % in the TU arm, 0 % in the ACT arm). A non-hematological toxicity (grade 3 nausea) was noted only in patients in the ACT arm (10 %). There was grade 3 rash (1.2 %) in a patient in the TU arm and grade 3 arrhythmia (1.3 %) in a patient in the ACT arm.

**Table 2:** Hematological (A) and non-hematological (B) toxicities

Toxicities	Grade 2 (%)	Grade 3 (%)	Grade 4 (%)
(A)			
*TU*			
WBC	8 (9)	0 (0)	0 (0)
Hb	2 (2)	3 (3)	0 (0)
T-bill	43 (49)	11 (13)	0 (0)
GOT	5 (6)	2 (2)	0 (0)
GPT	9 (10)	2 (2)	1 (1)
*ACT*			
WBC	12 (14)	3 (3)	0 (0)
Hb	7 (8)	0 (0)	–
T-bill	8 (9)	0 (0)	0 (0)
GOT	1 (1)	1 (1)	0 (0)
GPT	5 (6)	1 (1)	0 (0)
(B)			
*TU*			
Infection	0 (0)	0 (0)	0 (0)
Nausea/vomiting	7 (0)	0 (0)	–
Diarrhea	2 (0)	0 (0)	0 (0)
Arrhythmia	1 (1)	0 (0)	0 (0)
Thrombosis	0 (0)	0 (0)	0 (0)
Alopecia	0 (0)	–	–
Rush	2 (0)	1 (0)	0 (0)
*ACT*			
Infection	0 (0)	0 (0)	0 (0)
Nausea/vomiting	29 (36)	8 (10)	–
Diarrhea	1 (1)	0 (0)	0 (0)
Arrhythmia	1 (1)	1 (1)	0 (0)
Thrombosis	0 (0)	0 (0)	0 (0)
Alopecia	37 (46)	–	–
Rush	1 (1)	0 (0)	0 (0)

## Discussion

The decision to administer postoperative adjuvant drug therapy, which seeks to inhibit the recurrence of breast cancer, is often currently made based on the primary tumor’s subtype. Breast cancer is essentially categorized into four subtypes depending on the expression of ER, PgR, HER2, and Ki67 [2]. Endocrine drugs are given to patients with ER- and/or PgR-positive luminal tumors. Trastuzumab (a molecular-targeted agent) and an anticancer agent are both administered to HER2-positive patients. These strategies are tailor-made target therapies according to the prediction of efficacy of drugs. In addition to endocrine drugs, anticancer agents are often administered to patients with breast cancer expressing a high level of Ki67 [8, 9]. The individual determination of whether or not a tumor is sensitive to a drug is difficult, and despite this, anticancer agents are administered. Including anticancer agents is considered acceptable when patients have numerous lymph node metastases (irrespective of tumor subtype), if their cancer is ER- and/or PgR-positive and expressing a low level of Ki67. The validity and evaluation of Ki-67 are not definitive [10]. Both anthracycline and taxane are often administered sequentially for these patients despite the possibility that efficacy of these drugs is low. These classifications of breast cancer and administration of taxane and molecular drugs were widely in use after the current trial began.

At the beginning of this study, tamoxifen was administered as the standard therapy even if the patient was ER-negative. In light of current evidence, there is no doubt that tamoxifen has little efficacy in treating ER-negative breast cancer [11], though there are also no data indicating that the efficacy of anticancer agents will diminish if used in combination with tamoxifen. Thus, the results of this trial simply compared taking UFT for 2 years to taking AC to treat node-positive premenopausal breast cancer. Previous meta-analyses clearly revealed data indicating that AC therapy is more effective at preventing recurrence than CMF [12–16], but AC therapy has not been compared to oral fluoropyrimidine. The results of this trial indicated no difference between oral fluoropyrimidine and AC therapy in terms of prolonging survival in patients overall. AC therapy resulted in a longer recurrence-free survival (RFS) in only ER-negative patients. These results do not have a meaning for recent breast cancer treatment strategy, because of the insufficiency of patients recruitment and old adjuvant treatment design. However, this finding suggests that AC therapy has limited efficacy when treating node-positive breast cancer by administering tamoxifen as a postoperative adjuvant therapy to treat ER-positive breast cancer. This finding also suggests that administration of oral fluoropyrimidine alone may be sufficient in some cases. In fact, OS and PFS were similar between ACT and TU arm with ER-positive breast cancer. A potent anticancer agent, like anthracycline, may not be needed to treat ER-positive breast cancer even if it has lymph node metastasis.

The question of whether UFT is needed or if tamoxifen alone is sufficient remains. Results of the JCOG9401 study [17], which examined patients with postmenopausal breast cancer with lymph node metastasis during the same period as the current trial, may offer an answer. The study compared tamoxifen alone and ACT therapy to treat patients with node-positive breast cancer, and results indicated that ER-positive patients had a 5-year RFS of 59.3 % when given tamoxifen alone versus 76.9 % when given ACT therapy and a 5-year OS of 87.1 % when given tamoxifen alone versus 90 % when given ACT therapy. Patients in this trial who were given UFT+tamoxifen had a 5-year RFS of 74.5 % and a 5-year OS of 89 %. There was possibility of prognostic benefit of additional UFT for ER-positive node-positive patients. Thus, comparison of TU therapy to tamoxifen alone is needed. In Japan, a prospective clinical trial on adding S-1 to treat patients with ER-positive breast cancer after completion of standard chemotherapy is currently enrolling subjects (UMIN000003969).

No major differences were noted in ER-negative patients in either arm of this trial. That said, ER-negative patients had a 5-year OS and a 5-year RFS that was about 30 % shorter than the 5-year OS and 5-year RFS of ER-positive patients. Trastuzumab tends to be administered to patients with ER-negative breast cancer if they are HER2-positive [18], and taxane tends to be administered along with anthracycline if they are HER2-negative [14]. The regimens in this trial were inadequate to evaluate the appropriate adjuvant drugs for ER-negative patients with node metastases.

In terms of adverse events, a hematological event in the form of a grade 3 decline in the white blood cell count was noted only in patients in the ACT arm. In terms of non-hematological events, abnormal liver function was noted in patients in the TU arm and nausea was often noted in patients in the ACT arm. Results of this trial revealed numerous adverse events in patients in the ACT arm as a whole. Since the current dose of AC is higher than that used in this trial, UFT may be less damaging. However, results suggested that sufficient caution in abnormal liver function is necessary to use UFT for long time as adjuvant therapy. The current trial did not administer both endocrine therapy and chemotherapy concurrently. Previous data on such chemoendocrine therapy have highlighted the enhancement of adverse events and an increase in thrombosis in particular [19–21]. Neither group of patients in this trial had thrombosis/embolism. Existing data are from the USA and Europe, where thrombosis is more prevalent. These conditions may pose far less of a problem in Japan because of their different physique. Chemoendocrine therapy is ruled out based on current data from Europe and the USA, but there may be leeway for therapy selection depending on the patient.

This trial prospectively studied the usefulness of ACT therapy to treat patients with node-positive premenopausal breast cancer. This trial began prior to 2000, and modern standard adjuvant therapy was established during collecting patients for this trial. There were some issues with trial design and trial enrollment since the standard therapy changed substantially during trial enrollment. However, the times changed from an era of actively administering anticancer agents to every patient with breast cancer with lymph node metastasis to an era of selecting therapy by predicting drug efficacy. Postoperative adjuvant therapy with oral FU was the standard therapy in this trial, and a new appreciation for the efficacy of that therapy is developing. In this trial, ACT did not significantly prolong survival compared to TUFT, especially in ER-positive patients. Without a doubt, these findings pose clinical questions that should be answered when formulating a treatment strategy for postoperative adjuvant therapy. Further studies via prospective trials (which include those currently underway) are needed.
